# A Rare Case of Melanotic Schwannoma Occurred Intraosseous of Sacrum: A Literature Review

**DOI:** 10.1111/os.13606

**Published:** 2022-12-01

**Authors:** Xiaobo Yan, Keyi Wang, Nong Lin, Xin Huang, YanBiao Fu, Zhaoming Ye

**Affiliations:** ^1^ Department of Orthopedic Oncology The Second Affiliated Hospital of Zhejiang University Hangzhou China; ^2^ Key Laboratory of Motor System Disease Research and Precision Therapy of Zhejiang Province Hangzhou China; ^3^ Department of Pathology The Second Affiliated Hospital of Zhejiang University Hangzhou China

**Keywords:** Intraosseous, Melanotic schwannoma, Radiotherapy, Sacrum

## Abstract

**Background:**

Melanotic schwannoma is a rare tumor when it occurs in the sacrum. Though it is mostly classified as benign, the prognosis is unpredictable due to the possibility of recurrence and metastasis. Here, we reported a case of intraosseous of sacrum with good results and reviewed the literature.

**Case Presentation:**

A 33‐year‐old male patient complained of low back pain and was discovered to have an obstruction at S2. Following the necessary imaging diagnosis, we treated the patient with piecemeal excision in conjunction with extended curettage, and the frozen biopsy revealed that the tumor was melanotic schwannoma. The intraosseous portion of the lesion was curettaged using high‐speed drill to enlarge the edge of curettage, and piecemeal excision for lesion within the sacral canal. After surgery, the patient received total 56Gy radiotherapy and frequent follow‐up. After 15 months follow‐up, there was no evidence of recurrence, and the nerve function was normal.

**Conclusion:**

Melanotic schwannoma that occurs intraosseous of the sacrum is extremely rare and lacks typical clinical manifestations; however it can be identified through careful pathological and imaging diagnosis. Intralesional extended curettage combined with radiotherapy can achieve a good local control with a satisfactory clinical effect in this rare disease.

## Graphical abstract

We discussed our successful management of this challenging tumor with a focus on preoperative diagnosis, surgical approach, and histological consideration to provide guidance for future clinical management in melanotic schwannoma.

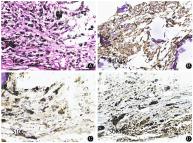

## Background

Melanotic schwannoma (MS) is a rare variant of schwannoma with capability of melanogenesis, which was first identified by Millar in 1932, accounting for only 1% of schwannoma.^1^ MS is frequently found in thoracic paraspinal region, retroperitonea, and extremities, but the sporadic occurrence of MS at unusual anatomic sites happens sometimes.^2^ For instance, it has been reported to occur in the spinal cord, sympathetic chain, cranial nerve roots, peripheral nerves, and the gastrointestinal tract, but it is extremely rare in the sacrum.^3^ Though MS is usually defined as benign tumors, there were some studies that suggest the prognosis of MS is unpredictable because of the propensity towards recurrence and metastasis compared to conventional schwannomas. Therefore, MS patients should be treated with complete resection of tumor or curettage with adjuvant treatment and regular follow‐up for local recurrence or metastasis to improve the overall survival. Here, we report a rare case of sacral MS which was successfully resected without postoperative complications in our institution, and provide a systematic review of the imaging, histopathology and prognostic outcomes of this MS patient. The purpose of this case report is to summarize the clinical features and imaging manifestations of such rare cases, as well as some pathological manifestations of common differential diagnosis, so as to aid in future clinical diagnosis.

## Case Presentation

A 33‐year‐old male patient presented to our clinic with 2 months of low back pain and a half‐month of right thigh numbness. The patient reported that both pain and numbness were activity related. When he presented, he did not have intermittent claudication. A CT scan was taken at an outside hospital, which revealed an obstruction at S2. The physical examination showed normal sensation and tenderness in his bilateral legs. At admission, the Lasègue test was conducted, and a negative result was obtained. Manual muscle testing is also employed and gastrocnemius muscular strength for both legs was scored at 5. Laboratory tests revealed that the patients' common blood cell count, C‐reactive protein level, erythrocyte sedimentation rate, and tumor markers were all within normal limits.

### Imaging Findings

**Fig. 1:**
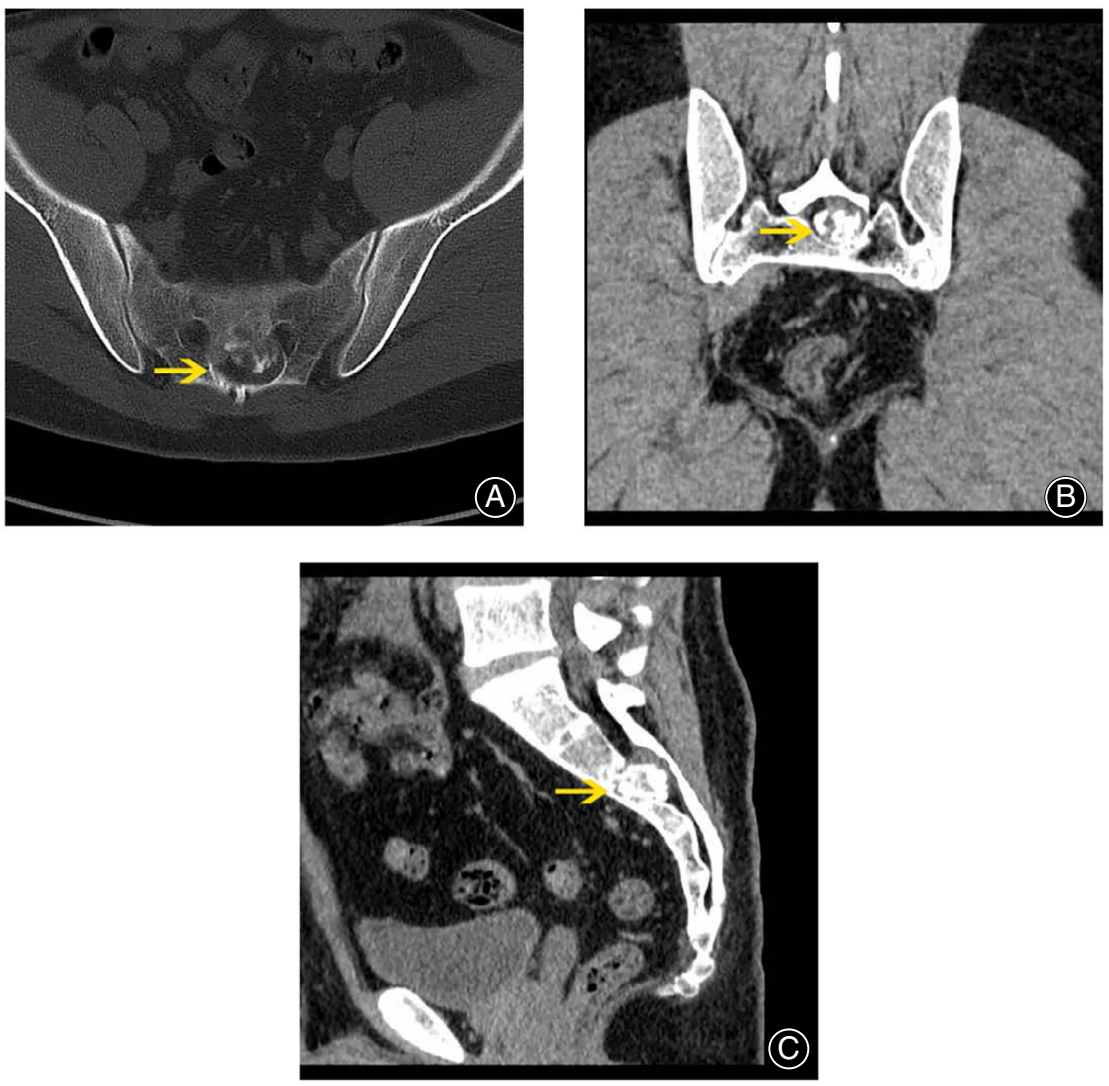
(A) The transverse CT scan showed the lesion in S2 level, an osteolytic lesion with calcified part; (B) the coronal CT scan showed the relationship between the tumor and sacral nerve; (C) the sagittal CT scan showed the lesion's location in AP direction

The CT scan showed an osteolytic lesion with local ossification at S2 level; the osteolytic lesion was located at the posterior of the vertebral column, and the calcified portion nearly reached the anterior of the vertebral column (Figure 1).

**Fig. 2:**
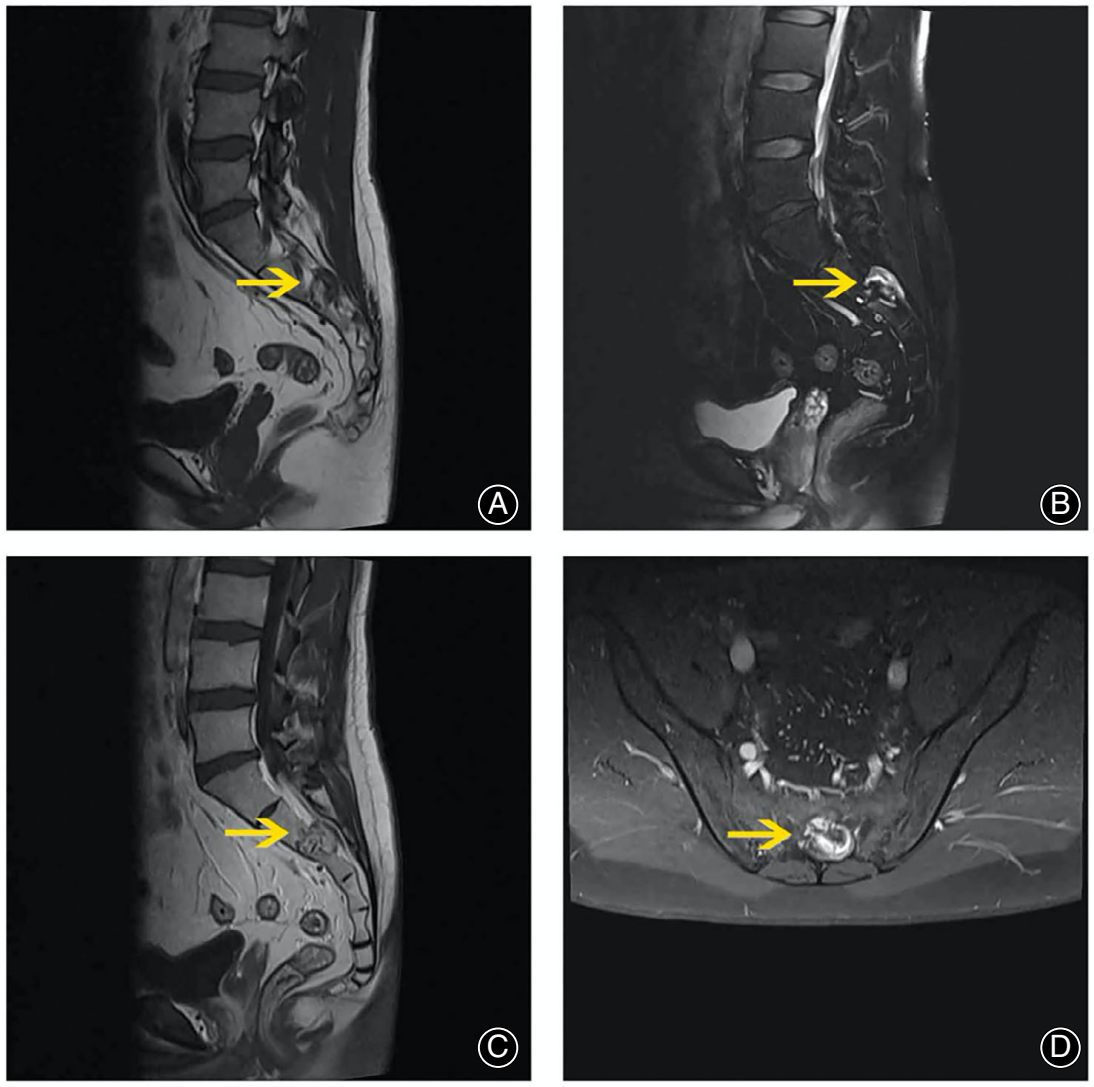
A destructive malignancy occupying spinal canal in the sacrum (arrow) was revealed in the sagittal view of lumbosacral spine MRI (A). The lesion displayed mixed signal intensity on T1‐weighted MRI (T1WI; A) and T2‐weighted MRI (T2WI; B, D). Contrast‐enhanced T1‐ weighted MRI (CET1; C) revealed a remarkable contrast enhancement

A magnetic resonance imaging scan of the sacral spine (MRI; Figure 2) revealed an irregularly shaped but well‐defined 2×2.5×2 cm mass in the spinal canal extending into the sacrum. On T1‐ weighted MRI, the lesion appeared hypointense in the spinal canal but hyperintense in vertebral part (T1WI; Figure 2A), and it was reversed on T2‐weighted MRI (T2WI; Figure 2B,D). Contrast‐enhanced T1‐weighted MRI (T1CE; Figure 2C) demonstrated remarkable contrast enhancement.

### Biopsy

We did not perform a biopsy in this patient due to the risk of nerve injury, and a benign tumor was first considered for surgical operation.

### Preoperative Diagnosis

The patient was considered to have a benign sacral tumor. The differential diagnosis for this tumor is extensive with osteoblastoma, enchondroma, schwannoma, or giant cell bone tumor being considered. The lesion occurred in the sacrum, which is a common location for giant cell bone tumor and schwannoma. The CT scan revealed an expanding mass to the sacral canal combined with osteogenic lesions and surrounded by a sclerotic ring. Such imaging manifestations are common in osteoblastoma and enchondroma, and in some cases of schwannoma, intralesional calcification can also present. Based on MR imaging, the lesion displayed mixed signal intensity on both T1‐weighted MRI and T2‐weighted MRI, but the gadolinium‐enhanced T1‐weighted MRI scan showed heterogeneous enhancement of the lesion. Such characteristic features can all be seen in osteoblastoma, enchondroma, schwannoma, and giant cell bone tumor.

### Surgical Treatments and Pathologic Findings

**Fig. 3:**
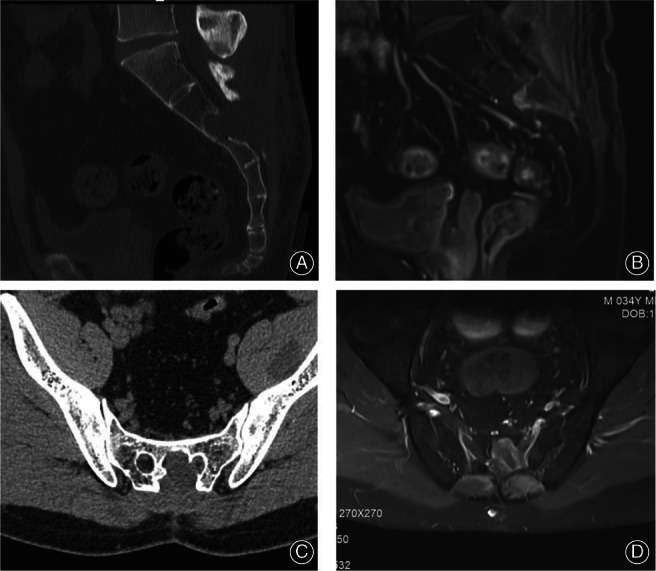
Both the sagittal postoperative CT scan and MRI showed extended surgical margin was achieved. (A and B); Both the transverse CT scan and MRI showed no sign of recurrence at the most recent follow up (C and D)

For this patient, extended curettage surgery was preferred considering the potentially benign nature of the tumor based on clinical diagnosis and inevitable neurological function deficit due to en‐bloc resection of the lesion. By opening the sacral canal, retracting and protecting the cauda equina, the lesion could be fully exposed for curettage and excision. The patient was put in a prone position and then S2 laminectomy was conducted. During the procedure, a firm, well‐shaped, encapsulated, and vascular‐rich blackish mass extending to the nerve sheath of the left S2 spinal nerve root was found. The dura was compressed and displaced to the right side of the spinal canal. A linear incision over the nerve sheath was conducted and a darkish hematoma was exposed. Due to its specific location, extended curettage surgery was performed for the intraosseous portion of the mass using high‐speed drill, and piecemeal excision for lesion located within the sacral canal. The specimen was then sent for a frozen biopsy, and result showed that the tumor was a melanotic schwannoma. Moreover, the edge of the mass tissue was sent for pathological assessment and a negative margin was obtained. The sagittal CT scan and MRI demonstrated an adequate surgical margin was achieved postoperatively (Figure 3A,B).

**Fig. 4:**
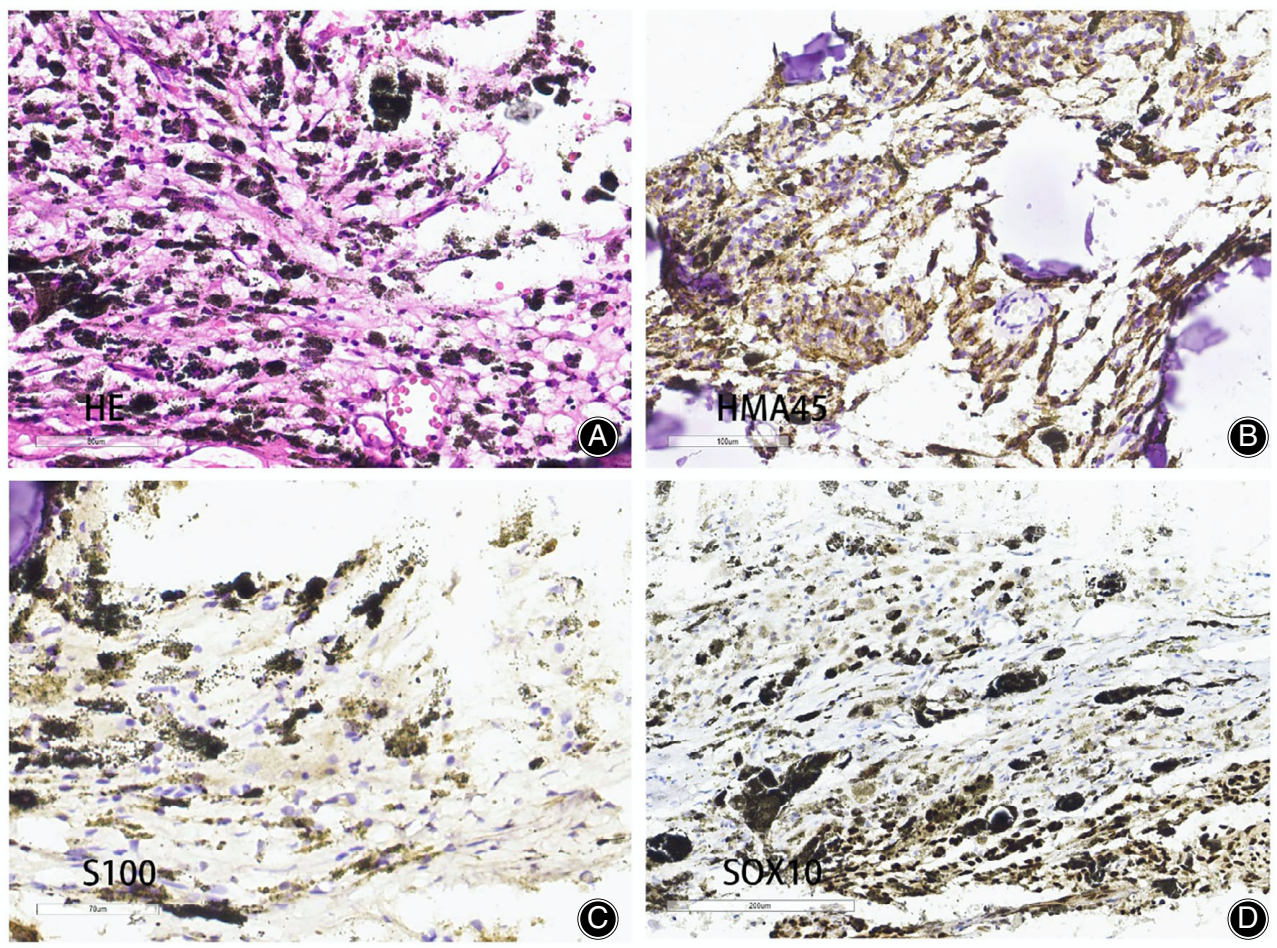
Histopathological examination revealed encapsulated cellular tumor with abundant eosinophilic cytoplasm and large vesicular nuclei. The cytoplasm contained a large number of coarse brown pigmented granules (A). Immunohistochemical staining showed HMB‐45 positive (B), S‐100 positive (C) and SOX‐10 partial‐positive (D)

Following the operation, the pathological specimens were subjected to routine hematoxylin‐eosin (HE) staining and immunological marker reexamination. Melanotic schwannoma was confirmed by specialized pathologists. Under the light microscopy, the tumor cells were spindle‐shaped, epithelioid, and arranged in fascicles, palisades, or nests under the light microscope. The nuclei were relatively uniform in size, and the nucleoli were not visible. Mitotic figures were uncommon, but abundance of melanin was observed. There was no obvious necrosis and only cytoplasm was found. Typical schwannoma tissue conformation can still be seen in some areas. The tumor cells were immunohistochemically positive for homatropine methylbromide (HMB‐45), S‐100 protein, partially positive SOX‐10, and negative for epithelial membrane antigen. The labeling index for Ki‐67 was approximately 5%–10% (Figure 4). The slides were also sent to the pathology department of UCLA School of Medicine, one of the collaborators of our hospital, for pathological consultation, and Dr. Sarah Dry reconfirmed this diagnosis.

### Follow‐Up

The patient received total 56 Gy radiotherapy but no chemotherapy after surgery. The first outpatient review was scheduled for 1 month postoperatively. After that, the patient was regularly followed up once every 3 months. His symptoms were obviously relieved and there was no sign of local recurrence at the most recent follow‐up, 18 months postoperatively (Figure 4C,D). According to the patient‐reported outcomes, postoperative complications such as neurological deficits, stabilization, or ambulatory issues were not observed.

## Discussion and Conclusions

Though schwannoma is a common intradural extramedullary spinal tumor, it's rare when it occurs in the sacrum. Different diagnoses of sacral primary tumors include chordoma, giant cell tumor, hemangioma.^4^, ^5^, ^6^ Melanotic schwannoma is a special kind of schwannoma which was first reported by Millar in 1932 as a “malignant melanotic tumor of sympathetic ganglion cells.” In 1961, Hodson described it as a form of schwannoma.^7^ In 1990, Carney reported the melanotic schwannoma as a different pathological lesion, classifying it as a component of Carney complex along with cutaneous lesions, endocrine tumors, and cardiac myxoma.^8^ To date, only seven cases of sacral MS have been reported in the literatures (Table 1). This table again demonstrates that while not all forms of MSs will metastasize or relapse, MS should be regarded as a distinctive malignant tumor due to its unpredictable behavior.^5^, ^9^, ^10^, ^13^

**TABLE 1:** The documented cases of MS occurred in sacrum

Case	Sex/Age	Primary site	Nerve Sacrifice	Surgical Excision	Recurrence	Metastasis	Follow‐up (month)	Status	Reference
1	M/48	sacrum	S2 nerve	complete	No	No	6	ANED	Yoshitaka Nagashima *et al*
2	F/41	sacrum	No	incomplete	No	No	72	ANED	Vallat‐Decouvelaere *et al*
3	F/26	S2	S2 nerve	complete	No	No	unknown	unknown	Killeen *et al*.
4	M/69	sacrum	unknown	unknown	No	No	1	ANED	Torres‐Mora *et al*
5	F/25	sacrum	unknown	unknown	Yes	Lungs, pleura and mediastinal lymph nodes	6	DOD	Torres‐Mora *et al*
6	M/30	S1	unknown	unknown	unknown	unknown	unknown	unknown	Torres‐Mora *et al*
7	M/60	sacrum	unknown	unknown	unknown	unknown	unknown	unknown	Torres‐Mora *et al*

Abbreviations: ANED, alive without evidence of disease; DOD, die of disease; F, female; M, male.

MRI is the main imaging examination to differentiate MS from other conventional schwannomas. In general, schwannomas located in spinal canal showed precontrast T1 isointensity and T2 hyperintensity than the spinal cord.^11^ Malignant melanomas showed hyperintense on T1‐ weighted MRI (T1WI) and isointense to hypointense on T2‐weighted MRI (T2WI).^12^ Because of the paramagnetic free radicals in melanin, the lesions of MSs are typically hyperintense on T1WI and hypointense on T2WI, whereas the conventional schwannomas tend to be the opposite.^13^ Both types of schwannomas can be enhanced on contrast‐enhanced images.^14^ MSs are typically surrounded by a thin fibrous membrane that might be infiltrated by tumor. Since MSs are sometimes characterized by local hemorrhage or necrosis,^8^ these lobulated, soft, firm, blackish, or brownish masses indicate an invasive nature and growth pattern. Due to their potential aggressiveness, MSs are difficult for both experienced surgeons and radiologists to distinguish from other malignant bone tumors based solely on preoperative conventional imaging. In our case, the lesion was mixed‐intense on T1WI and hyperintense on T2WI, indicating a typical form of conventional schwannomas. There was no obvious edema in sacrum, and CT revealed a sclerosis border. So, without a biopsy, we diagnosed it as a conventional schwannoma.

18F‐fluoro‐deoxy‐glucosepositron‐emission‐tomography (18F‐FDG PET) is a remarkable tool in assessing the biological and histological characteristics as well as malignant capacity of the tumors prior to surgery, and therefore, is validated in assisting the prediction of patient prognosis.^15^ In 2008, Eary *et al*. reported cases of 238 patients with sarcoma and concluded that SUVmax is applicable in predicting prognosis. In our case, we did not order the 18F‐FDG PET for this patient, so we failed to make any assumptions based on it.

The biopsy was a useful method for differentiating MSs from other primary tumors. However, there is a risk of complications such as infection, wound hemorrhage, nerve injury, and other related problems after the procedure. As a result, we did not plan the core needle biopsy for this patient, and instead performed an intralesional curettage surgery. During the surgery, we discovered that S2 nerve root was the source of the tumor, and the sacrum was infiltrated by it. The border was not as clear as it appeared on CT, and there appeared to be a black spot in cancellous bone, so we used high speed drill to conduct extended curettage to remove the intraosseous portion, and piecemeal excision for lesion within the sacral canal.

The MSs are distinguished by the accumulation of melanin in neoplastic cells and the formation of the cytoplasmic melanosome. Most MSs are well‐defined or encapsulated in a round or oval shape with cut section being black, brown, or gray. They typically range in diameter from 0.5 to 26 cm, with the majority being larger than 5 cm. Histologically, MSs can be classified into two types: conventional melanotic schwannoma (CMS) and psammomatous melanotic schwannoma (PMS). CMSs are typically composed of spindle‐shaped and epithelioid cells arranged in bundles or interlaced fascicular pattern, with nuclear palisading. Verocay bodies are uncommon in CMS, but pigmented granules can be seen in cytoplasm of most tumor cells. The nucleus can sometimes be obscured by excessive pigmentation. For PMSs, there are some stratified calcified globules, most of which are focal lesions with a round or oval shape. In approximately 60% of PMSs cases, intracytoplasmic vacuoles can be seen within the tumor cell, mimicking mature adipose tissue if there are only few of them, and tumor reported in this case fall into PMS category.

This case should be carefully differentiated from the following three types of tumors: pigmented neurofibroma (TPN), metastatic malignant melanoma (MMM), and soft tissue clear cell sarcoma (STCCS). TPN often occurs in the dermis or subcutaneous tissue of the head or neck, or lower extremity such as buttocks or calves. With an undefined margin, the malignancy typically spreads along connective tissue and fat lobular septa and can surround skin appendages, etc. Small‐ to medium‐sized spindle or oval‐shaped tumor cells are sparsely arranged. The interstitium is composed of uniform collagen fibers that proliferate to form carrot‐like collagen bundles. Pigment cells are displayed within the cells in a dendritic manner. Angiohyalinosis is uncommon in neurofibroma, and S‐100 positive reaction are also less commonly seen in neurofibroma in contrast to schwannoma. MMM is closely related to the skin as nearby skin is the most common site for its regional metastasis. Patients with this malignancy are typically middle‐aged, generally over 40 years old and with a history of malignant melanoma. The pathology of MMM is characterized by obvious pleomorphism and cytologic atypia as well as large eosinophilic nucleolus, nuclear groove, intranuclear inclusions, and prominent mitotic figures. Unlike TPN and MMM, STCCS are most commonly seen in the distal end of extremity, particularly the feet and ankles, followed by the knees, thighs, hands, and forearms, and, on rare occasions, the torso, head and neck, intestines, and kidneys. The location of STCCS is deep‐seated, often connecting with tendons and aponeuroses, and can invade throughout subcutaneous tissues. The tumor cells are densely arranged in bundles, nests, or sheets, separated by thin or dense fibrous connective tissue. Often polygonal, oval, or spindle‐shaped, they are prominent by clear cytoplasm, round or oval‐shaped nuclei and eosinophilic nucleoli. Mitotic figures were not common and multinucleated giant cells can be seen occasionally. Immunohistochemical staining for S‐100, HMB‐45, Melan‐A, and NSE‐7 showed positive results in STCCS cells. However, in this case, the specimen shows spindle cells arranged in sheets and short fascicles, with heavy deposition of melanin pigment that largely obscures the nuclear details of most cells. Moreover, S‐100 and SOX‐10 immunostains are strongly and diffusely positive. By combining the aforementioned characteristics, our case can be differentiated from TPN, MMM, and STCCS.

Surgery is the first‐line treatment option, and a complete resection of tumor with clear margins is recommended. In 2003, Klimo *et al*.^16^ reported in their publication that the sacral schwannoma can be classified into three types based on their position relative to the sacrum. For type I tumors which are mostly confined to the sacrum, complete resection can be achieved *via* posterior approach alone; unlike type I tumor, type II sacral tumors originate in the sacrum but metastasize towards the presacral and subcutaneous region, and consequently combined anterior–posterior approach is necessary; type III tumor is located in the presacral or retroperitoneal alone, which an anterior approach is appropriate. Though some authors reported satisfying results without local recurrence can be obtained *via* piecemeal surgery with sacral nerve roots being preserved,^17^ en‐bloc resection of the tumor was still recommended in most literature of MS for its excellent local control. However, given the origin, location, as well as the extent of intraosseous invasion of the MS, it is difficult to ensure a complete tumor removal while maintaining nerve integrity, and thus presumably pose a great impact on the patients' quality of life. In 2020, Yoshitaka *et al*.^13^ reported a case of an en‐bloc resection of sacral MS along with left S2 nerve root with no sign of recurrence up to 6 month follow‐up, yet he did not mention how such neurological deficits might affect patient's postoperative quality of life including physical/mental health, bowel function, etc. In our case, we did not conduct biopsy prior to surgery in order to avoid potential risk of a series of site‐related complications, and to achieve tumor resection while preserving S2 function, extended curettage in combination of piecemeal excision was performed *via* posterior approach. The patient was regularly followed up once every 3 months and has shown no signs of recurrence or metastasis up to this point.

Adjuvant therapy, such as chemotherapy or radiotherapy, is not well‐defined yet, but for cases with aggressive histology (i.e., >2 mitosis/10 hpf), adjuvant therapy is recommended.^9^ For tumors without clear margins, adjuvant radiation is helpful in decreasing the risk of local recurrence and metastasis. Besides, the introduction of various types of immunotherapies provides patients with additional treatment options. It should be noted, however, that PD‐L1 expression was immunonegative in all previous cases.^18^ Although in 2021, Charles C. *et al*. reported in one case of retrocaval MS that the use of pembrolizumab can lead to significant improvement in pain control and tumor stability, they emphasized the necessity to assess its applicability to general MS.^19^ Despite the fact that MSs have generally been regarded as benign neoplasms, growing evidence suggests that they are distinctive malignant tumors with the potential to metastasize and relapse due to their unpredictable behavior. According to Vallat‐Decouvelaere,^20^ the metastatic rate in patients with MS was up to 26%. Zhang *et al*.^15^ reported a 9.1% metastases rate in MS and 18.2% local recurrence rate after resection. However, Torres‐Mora *et al*. revealed a 35% local recurrence and a 44% metastasis rate in MS patients, indicating a significantly more aggressive nature. Since MS patients tend to display late local recurrence and metastases, long‐term follow‐up is necessary despite of the absence of malignant histological characteristics.

### Conclusions

Melanotic schwannoma is a rare variant of schwannoma with aggressive and malignant nature. Due to its rarity, MS is difficult to diagnose before surgery, and it is necessary for intra‐operative histological analysis to guide the extent of resection. So far, very few cases of intraosseous sacral MS have been reported. In this case, intralesional resection combined with radiotherapy not only avoids unnecessary neurological deficits but also achieves a good local control with a satisfactory clinical effect, which provide an alternative surgical management for MS. From our point of view, early and regular follow‐up *via* CT and MRI are advisable, and a long‐term follow‐up and observation of the patient is also required before a more definitive statement can be made concerning the prognosis of this rare case.

## Authors' Contributions

All authors contributed to the study conceptualization and design. Material preparation and data collections were performed by Xiaobo Yan, Nong Lin, and Xin Huang. Data integration was performed by Keyi Wang. Pathology diagnosis was conducted by Yanbiao Fu. The first draft of the manuscript was written by XBY and approved by Zhaoming Ye. Keyi Wang was responsible for review and editing. All authors commented on previous versions of the manuscript and all authors read and approved the final manuscript.

## Conflicts of Interest

The authors declare that they have no competing interests.

## Ethics Approval

This study was approved by the Human Research Ethics Committee of the Second Affliated hospital of Zhejiang University School of Medicine. 2022‐0031. The written informed consent to participate was obtained from the patient.
